# Valgus stress radiographs have a minor additional value in detecting lateral cartilage wear when selecting patients for medial partial knee replacement

**DOI:** 10.1007/s00402-023-05187-0

**Published:** 2024-01-12

**Authors:** Stephan J. van Langeveld, Stein J. Janssen, Iris Koenraadt-van Oost, Maaike P. Terra, Rutger C. I. van Geenen

**Affiliations:** 1 Department of Orthopaedic Surgery, FORCE (Foundation for Orthopaedic Research Care and Education), Amphia Hospital, Molengracht 21, 4818CK Breda, The Netherlands; 2 Department of Orthopedic Surgery and Sports Medicine, Academic Medical Center, Amsterdam University Medical Center, Meibergdreef 9, 1105 AZ Amsterdam, The Netherlands; 3 Department of Radiology, Amphia Hospital, Molengracht 21, 4818CK Breda, The Netherlands

**Keywords:** Medial knee osteoarthritis, Valgus stress radiograph, Lateral joint space width

## Abstract

**Introduction:**

A valgus stress radiograph, in addition to the weight-bearing fixed flexion posteroanterior radiograph (e.g., Rosenberg), is deemed useful to assess lateral cartilage wear by measuring lateral joint space width (JSW) in patients with medial knee osteoarthritis. This study aimed to assess: (1) the difference in measured lateral JSW between the Rosenberg and the valgus stress radiograph, and (2) the ability of the valgus stress radiograph to detect lateral cartilage wear (indicated by joint space narrowing) in patients where the Rosenberg radiograph showed full thickness cartilage (i.e., the additional value).

**Materials and methods:**

The Rosenberg and valgus stress radiographs, obtained between January 1st 2018 and December 31st 2018, of 137 patients with medial knee osteoarthritis prior to total or partial knee replacement were retrospectively collected. The lateral JSW was measured at its midpoint (midJSW) and minimum (minJSW). The differences were tested with a paired-sample *t* test. The valgus stress radiograph was considered to have an additional value if: (1) JSW ≥ 5 mm on the Rosenberg radiograph, (2) JSW < 5 mm on valgus stress radiograph, and (3) > 2 mm less JSW on the valgus stress than on the Rosenberg radiograph.

**Results:**

The mean differences in lateral JSW between the Rosenberg and valgus stress radiographs were 0.53 mm (SD = 1.0 mm, *p* < 0.001) for midJSW and 0.66 mm (SD = 1.1 mm, *p* < 0.001) for minJSW with both values being lower on the valgus stress radiograph. The valgus stress radiograph was of additional value in 4–6% of the patients.

**Conclusions:**

Although the valgus stress radiograph shows more lateral JSW narrowing compared to Rosenberg radiograph, it only has an additional value in 1 out of 17–25 patients with medial osteoarthritis. We, therefore, recommend a Rosenberg radiograph as routine radiographic assessment and only use an additional valgus stress radiograph in case of discrepancy between clinical and radiological findings.

## Introduction

Medial partial knee replacement (PKR) is an alternative to total knee replacement (TKR) for isolated medial compartmental knee osteoarthritis [1]. Various studies reported better clinical outcomes of PKR compared to TKR [1–3]. However, most comparative studies report higher revision rates after PKR as compared to TKR, which is reason for debate [4]. These higher revision rates are potentially explained by a lower threshold for revision after PKR compared to TKR, a lower surgical caseload which is related to higher revision rates, and inadequate patient selection for PKR [5–8].

The traditional indications for PKR, suggested by Kozinn and Scott, included single compartmental degenerative osteoarthritis, absence of patellofemoral osteoarthritis and chondrocalcinosis, age over 60 years, weight below 82 kg, and low activity demands [9]. However, the indication for PKR has continued to expand based on extensive research. Patellofemoral osteoarthritis, chondrocalcinosis, the patients’ age, weight or activity level do not affect outcome after PKR and are therefore no longer considered contra-indications [10]. Currently, PKR is indicated for patients with complete cartilage wear in the medial compartment and full thickness cartilage in the lateral compartment and a sufficient anterior cruciate and medial collateral ligament [11].

For patient selection, radiographic imaging is important to identify the degree of cartilage wear, which is measured by the joint space width (JSW), in both tibiofemoral compartments [12]. In clinical practice, the weight-bearing fixed flexion posteroanterior knee radiograph (e.g., Rosenberg) is recommended for assessment of the lateral JSW [13, 14]. However, as medial osteoarthritis is generally accompanied by varus deformity, overestimation of the lateral JSW is expected in weight-bearing radiographs [15]. Valgus stress radiographs are, therefore, deemed useful to improve assessment of lateral compartment cartilage thickness and might thereby help increase accuracy of patient selection for PKR versus TKR [16, 17].

Studies that directly compare the Rosenberg and valgus stress radiographs are scarce. Moreover, the difference found in JSW measured between the Rosenberg and valgus stress radiographs are very small (< 1 mm) [18]. Yet, the added value of valgus stress radiographs in patients with (suspected) medial knee osteoarthritis remains unclear and would be valuable to assess. This study therefore aimed to determine the clinically relevant value of the valgus stress in addition to the Rosenberg radiograph. First, by assessing the difference in measured lateral JSW between the Rosenberg and the valgus stress radiograph and the ability of the valgus stress radiograph to detect lateral cartilage wear in patients where the Rosenberg radiograph showed full thickness cartilage (i.e., the additional value).

## Methods

### Patient population

**Fig. 1:**
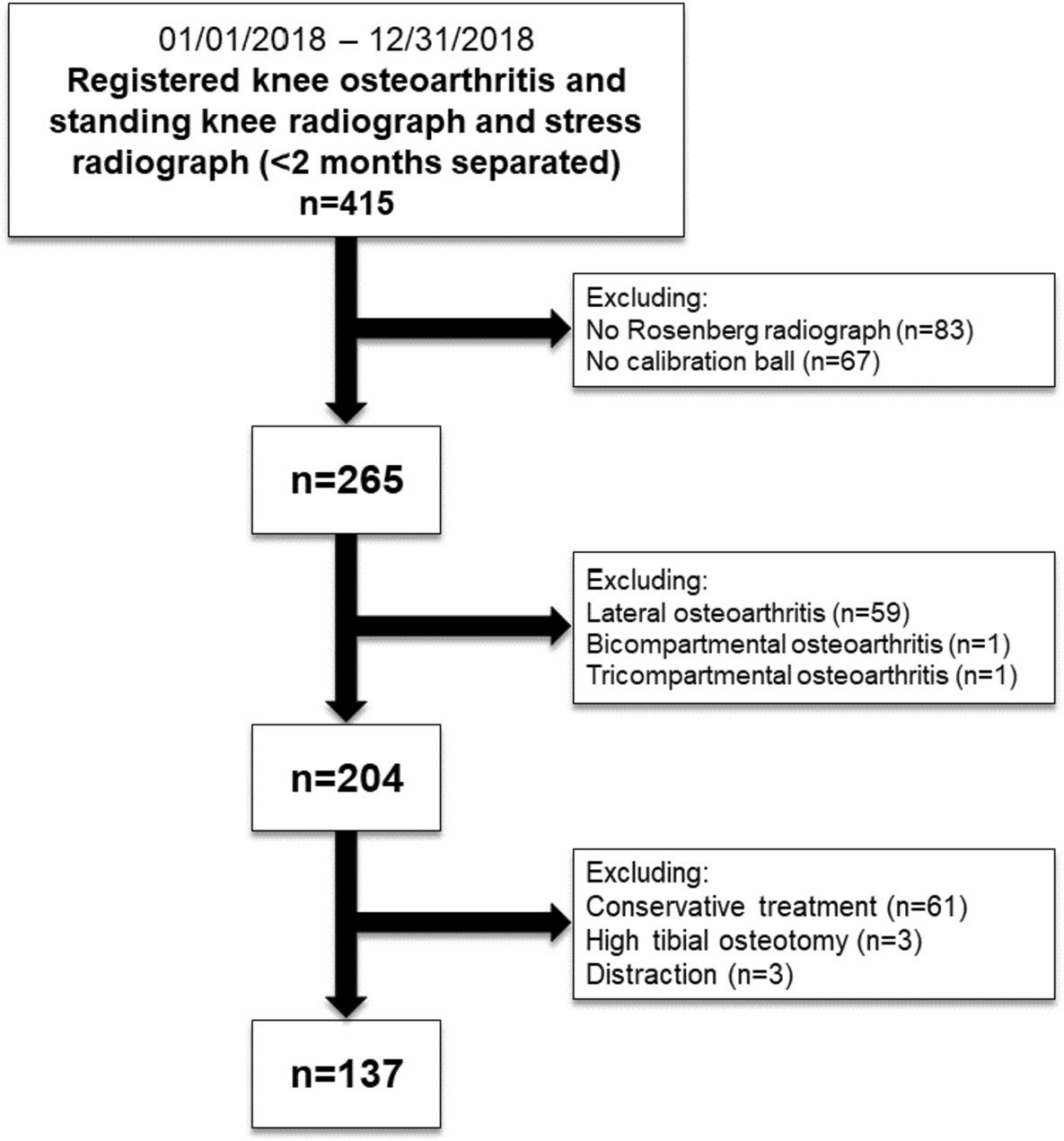
Flow chart

All consecutive patients with (suspected) medial knee osteoarthritis that underwent both Rosenberg and stress radiographs (within 2 months) in 2018 (between January 1st and December 31st) at a single urban center (“Name” Hospital, “City”, “Country”) were included in this retrospective cross-sectional study. All patients underwent subsequent knee replacement (partial or total knee replacement). All patients registered with the ICD-10-M code for knee osteoarthritis and a billing code for the standing knee radiograph and the stress radiograph with a maximum of 2 months separated were extracted from the electronic patients’ files. Exclusion criteria were patients that received a standing knee radiograph other than a Rosenberg radiograph, no calibration sphere included on either the Rosenberg or the valgus stress radiograph, patients diagnosed with inflammatory diseases (e.g., rheumatoid arthritis), patients diagnosed with lateral, bi- or tricompartmental osteoarthritis, and patients that received a conservative treatment or operative treatment other than PKR and TKR (Fig. 1).

### Imaging techniques

Both Rosenberg and valgus stress radiographs were executed according to a protocol by dedicated radiographers. For calibration a 30 mm calibration steel sphere was placed besides the knee joint.

The Rosenberg radiographs were obtained with the patient full weight-bearing evenly divided on both feet and the patella as close to the image detector as possible. The knee was in 45° flexion with equally divided distance between the detector and lower and upper leg. A posteroanterior X-ray beam was centered at the knee joint line with a 10° caudal inclination. The acquired image was then evaluated and approved if the joint space was projected free, the tibial plateau was horizontally aligned, and the patella was projected centrally over the femur.

The valgus stress radiographs were obtained with the patient in supine position with 20° flexion of the knee created by a supportive 20° wedged pillow. Valgus force was applied through a stress device (Telos Stress Device, Telos Arzt- und Krankenhausbedarf GmbH, Germany) at a standardized force of 15 Newton located laterally at the height of the joint line. Using fluoroscopy, the radiographer evaluated and approved the image according to the same criteria as for the Rosenberg radiography. The knee position or X-ray tube direction was adjusted when necessary.

### Radiograph measurements

The radiographs were analyzed through a picture archiving and communication system (JiveX DICOM viewer, VISUS Health IT GmbH 2020, Germany). Quantitative measurements were performed using a digital caliper and expressed in millimeters with one decimal. All radiographs were analyzed by an orthopedic trainee. The Rosenberg and stress radiographs were measured on two separate occasions to minimize recognition. About two-thirds of the radiographs were also assessed by a second orthopedic trainee to assess interobserver agreement.

**Fig. 2:**
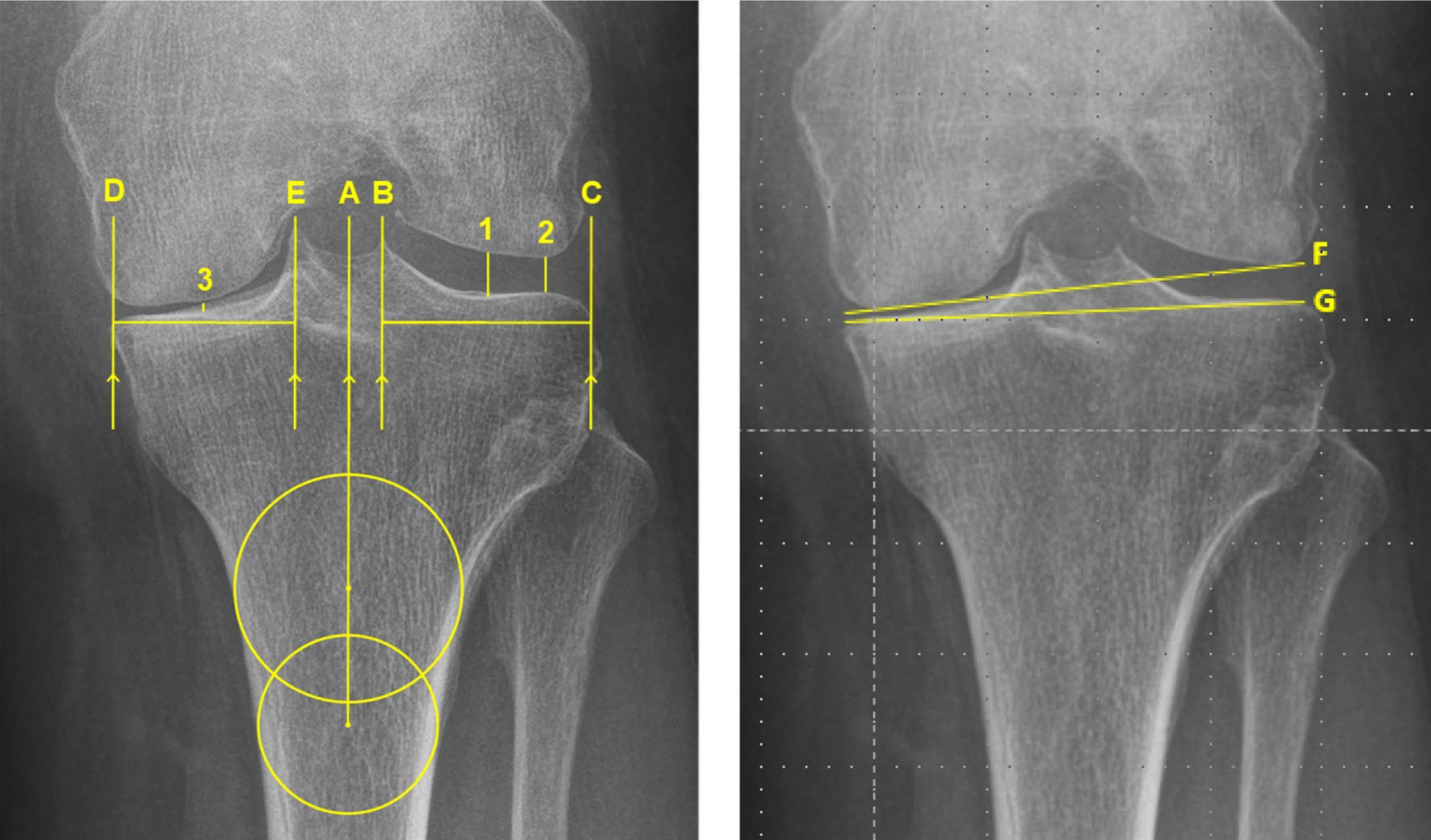
Radiographic measurements of the joint space width and intermargin distance. The joint space width measurements are similarly performed on the Rosenberg and valgus stress radiographs. The intermargin distance is only measured on the Rosenberg radiographs. *A* tibial axis, *B* line at the lateral intercondylar tubercle, *C* line at lateral aspect of the tibial plateau, *D* line at the medial aspect of tibial plateau, *E* line at the medial intercondylar tubercle, *F* line tangential to the distal femoral condyle, *G* line tangential the tibial plateau, *1* the midpoint joint space width, *2* the perceived minimum joint space width, *3* intermargin distance at the midpoint

The lateral JSW was measured parallel to the tibial axis at the midpoint of the lateral compartment (midJSW) and at the perceived minimum lateral joint space width (minJSW). For the midJSW, the rater selected the distance equidistant between the lateral aspect of the tibial plateau and the apex of the lateral intercondylar tubercle. For the minJSW, the rater selected what appeared to be the smallest distance between femur condyle and tibial plateau in the lateral compartment (Fig. 2) [19, 20].

As imperfect alignment is related to measurement (in)accuracy of the JSW, radio-anatomic alignment of the medial tibial plateau was evaluated on the Rosenberg radiographs by measuring the intermargin distance (distance between anterior and posterior tibial margin [IMD]) at the midpoint of medial tibiofemoral compartment (Fig. 2) [21, 22]. As fluoroscopy was used to align the knee in valgus stress radiographs, the assessment of the IMD in these radiographs was deemed unnecessary. To enable assessment of the influence of radio-anatomic alignment, the data was divided in a group with low IMD (i.e., well aligned) and high IMD (i.e., poor aligned) according to the populations IMD median.

Additionally, the joint line convergence angle (JCLA) was collected for the Rosenberg and valgus stress radiographs to assess the varus deformity correctability and sufficiency of the medial collateral ligament. The joint line convergence angle was measured by the angle formed between a line tangential to the distal femoral condyle and the tibial plateau with positive values representing an intra-articular varus and negative values an intra-articular valgus malalignment (Fig. 2). Moreover, the varus deformity correctability and therefore sufficiency of the medial collateral ligament during the preoperative physical examination was collected from the patient electronic record. The varus deformity was defined correctable and the medial collateral ligament sufficient if the surgeon stated ‘a redressable varus deformity’ and ‘sufficient collateral ligaments’.

### Statistical analyses

Normally distributed variables are presented as mean with standard deviation (SD) and non-normally distributed variables as median with range.

The differences in midJSW and minJSW between Rosenberg and valgus stress radiographs were calculated using a paired-sample *t* test. A Bland–Altman analysis and scatterplot were used to calculated and assess the mean difference and its 95% limits of agreement (mean difference ± [1.96 SD]) between the lateral JSW on Rosenberg and valgus stress radiographs [23]. As the JSW was normally distributed on both Rosenberg and valgus stress radiographs, Pearson’s correlation was used to assess the correlation between JSW measurements. The strength of the relationship was interpreted as poor (less than 0.30), fair (0.30–0.59), moderately strong (0.60–0.79), and very strong (more than 0.8) [24]. To consider clinical impact of the found difference, the percentage of JSW measured > 1 mm and > 2 mm lower on the valgus stress compared to the Rosenberg radiographs was calculated. The valgus stress radiograph was considered to have an additional value if the following criteria were met: (1) JSW ≥ 5 mm on the Rosenberg radiograph, (2) JSW < 5 mm on valgus stress radiograph, and (3) > 2 mm lower JSW on the valgus stress than on the Rosenberg [25]. The difference of > 2 mm was chosen to maintain a clinically relevant difference that also accounts for measurement errors. The ‘additional value’ of the valgus stress radiograph was reported as percentage of the total study population.

To assess interobserver reliability of the measurements, a subsample of the radiographs (about two-thirds of the data, 65%, *n* = 89) was analyzed by a second rater [26]. The interobserver agreement was calculated using the intraclass correlation coefficient (ICC) and presented with 95% confidence interval (95% CI). The strength of agreement was interpreted as poor (less than 0.50), moderate (0.50–0.74), good (0.75–0.89), and excellent (0.90–1.0) [27].

Significance was defined as a two-tailed *p* value below 0.05. All data were analyzed using IBM SPSS statistics software (version 25.0, Armonk, NY: IBM Corp). All figures are illustrated using GraphPad Prism (version 8.4.3, San Diego, CY: GraphPad Software).

## Results

A total of 137 knees of 137 patients were included in this study (Fig. 1). The mean age was 68 years (SD = 8.4 years) and 53% (*n* = 73) were female. The mean body mass index at time of knee replacement was 30 kg/m^2^ (SD = 5.4 kg/m^2^). 106 patients (77%) received a PKR and 31 (23%) total knee replacement. In 17 patients that were scheduled for a PKR, intra-operatively conversion to TKR was decided upon based on anterior cruciate ligament insufficiency (*n* = 5) and found lateral osteoarthritis upon direct visual inspection (*n* = 12). In 7 (5%) patients that were scheduled for TKR, the surgeon’s decision for TKR was based on increased lateral joint space narrowing on the valgus stress compared to the Rosenberg radiograph. The median (range) days between the Rosenberg and valgus stress radiographs was 16 days (0–62 days).

### Rosenberg radiographs

The mean lateral JSW was 6.6 mm (SD = 1.4 mm) for midJSW and 5.9 mm (SD = 1.3 mm) for minJSW on the Rosenberg radiographs. The interobserver agreement was found to be good for both midJSW (ICC = 0.80 [95% CI 0.69–0.87], *p* < 0.001) and minJSW (ICC = 0.75 [95% CI 0.59–0.85], *p* < 0.001). The mean interobserver measurement differences were 0.72 mm (95% CI 0.02–0.1.8 mm) for midJSW and 0.77 mm (95% CI 0.03–1.9 mm) for minJSW.

### Valgus stress radiographs

The mean lateral JSW was 6.1 mm (SD = 1.2 mm) for midJSW and 5.2 mm (SD = 1.1 mm) for minJSW on the valgus stress radiographs. The interobserver agreement was found to be excellent for both midJSW (ICC = 0.92 [95% CI 0.88–0.95], *p* < 0.001) and minJSW (ICC = 0.95 [95% CI 0.92–0.96], *p* < 0.001). The mean interobserver measurement differences were 0.45 mm (95% CI 0.03–1.2 mm) for midJSW and 0.33 mm (95% CI 0.05–0.96 mm) for minJSW.

### Rosenberg versus valgus stress radiographs

**Fig. 3:**
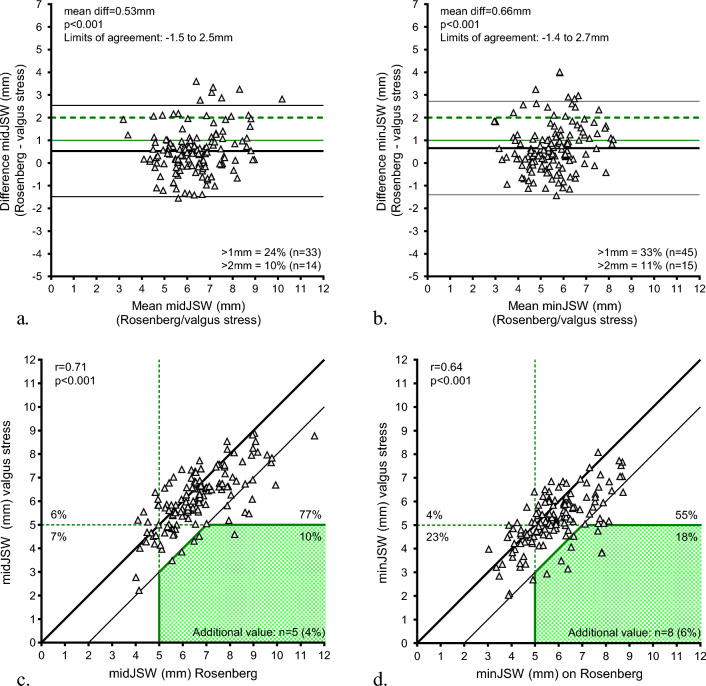
The level of agreement and relationship between Rosenberg and valgus stress radiographs. Bland–Altman plot of agreement in JSW between Rosenberg and valgus stress radiographs at midJSW (**a**) and minJSW (**b**) with the mean difference, limits of agreement, and > 1 mm (green solid line) and > 2 mm (green dotted line) lower JSW measured on the valgus stress radiograph. Pearson correlation plots between Rosenberg and valgus stress radiographs at midJSW (**c**) and minJSW (**d**) with the addition value (green shaded area) defined as: JSW ≥ 5 mm on the Rosenberg and < 5 mm on the valgus stress radiograph with > 2 mm difference (thin black line) between the measured values. *midJSW* midpoint joint space width, *minJSW* minimum joint space width, *JSW* joint space width

The comparison of the lateral JSW between the Rosenberg and valgus stress radiographs showed a mean difference of 0.53 mm (SD = 1.0 mm) for midJSW and 0.66 mm (SD = 1.1 mm) for minJSW with both measurements narrower on the valgus stress radiographs (*p* < 0.001). The estimated limits of agreement were – 1.5 to 2.5 mm for midJSW and – 1.4 to 2.7 mm for minJSW (Fig. 3a, b).

The correlation between the Rosenberg and valgus stress radiographs measurements was found to be moderately strong for midJSW (*r* = 0.71 with *p* < 0.001) and minJSW (*r* = 0.64 with *p* < 0.001).

The lateral JSW on the valgus stress radiographs was > 1 mm narrower in 24% of the patients at midJSW and in 32% at minJSW as compared to Rosenberg radiographs. The lateral JSW on the valgus stress radiographs was > 2 mm narrower in 10% at midJSW and in 11% at minJSW compared to Rosenberg radiographs. The calculated ‘additional value’ of the valgus stress radiograph was 4% at midJSW and 6% at minJSW (Fig. 3c, d).

### Influence of radio-anatomic alignment on joint space measurements

The median intermargin distance (IMD) was 2.4 mm (range = 0.09–9.2 mm). The mean difference in JSW for the low IMD (IMD < 2.4 mm) was 0.39 mm (SD = 0.93 mm) for midJSW and 0.53 mm (SD = 0.92 mm) for minJSW with both *p* < 0.001. The mean difference (SD) increased for the high IMD (IMD ≥ 2.4 mm), as these were 0.67 mm (1.1 mm) and 0.79 mm (1.2 mm) with *p* < 0.001. A JSW of > 2 mm narrower on the valgus stress was less frequently found in the low IMD compared to the high IMD (4% versus 16% at midJSW and 4% versus 17% at minJSW) (Appendix 4a–d). The strength of relationship was stronger in low IMD compared to high IMD, namely: 0.79 (*p* < 0.001) versus 0.62 (*p* < 0.001) for midJSW and 0.75 (*p* < 0.001) versus 0.51 (*p* < 0.001) for minJSW (Appendix 5a–d). The ‘additional value’ of the valgus stress radiograph was lower in low IMD compared to high IMD for both midJSW (3% versus 4%) and minJSW (3% versus 9%) (Appendix 2a–d).

### Varus deformity correctability

The mean JCLA was 4.8° (SD = 2.0° and range = – 1.1 to 9.3°) on the Rosenberg and – 0.36° (SD = 1.6° and range = – 3.4 to 4.2°) on the valgus stress radiographs (*p* < 0.001). For 6 (4%) knees the valgus stress radiograph showed a JCLA of > 3° varus. The varus correctability during preoperative physical examination was not specified in 18% (*n* = 25) of the patients; in the remainder (82%, *n* = 112), surgeons described a correctable varus deformity to a clinically acceptable knee axis with a sufficient medial collateral ligament.

## Discussion

In this study, the value of the valgus stress radiograph in addition to the Rosenberg radiograph for assessment of lateral cartilage wear, indicated by joint space narrowing, in patients with medial osteoarthritis was investigated. We found that the valgus stress radiograph measures on average a 0.53–0.66 mm lower lateral JSW compared to the Rosenberg radiograph. Overall, in 10–11% of the patients, the JSW was measured more than 2 mm lower on the valgus stress radiograph than on the Rosenberg radiograph. However, the valgus stress radiograph appeared to have an additional value (i.e., detecting joint space narrowing which was absent on the Rosenberg radiograph with a difference in JSW of more than 2 mm) in only 4–6% of the patients with medial osteoarthritis.

As progression of osteoarthritis is a frequent mode of failure in PKR, preoperative radiographic assessment of the lateral compartment cartilage is important when considering PKR [28]. The assessment of the lateral JSW is generally recommended to detect cartilage wear, indicated by joint space narrowing. Studies suggest that a JSW of 5 mm should be used to distinguish between full thickness cartilage (JSW ≥ 5 mm) and cartilage wear (JSW < 5 mm) [25, 29]. Due to the varus deformity with medial compartment osteoarthritis and subsequently overestimated measurements on the lateral side, the valgus stress radiograph is theoretically preferable for measuring the lateral JSW [15]. Hamilton et al. found in the validation of their treatment algorithm the valgus stress radiographs in addition to weight-bearing anteroposterior radiographs identify an extra 10% of missed lateral compartment osteoarthritis [16]. Moreover, studies have demonstrated that the valgus stress radiograph shows more narrowing of the JSW compared to conventional weight-bearing radiographs and that, if the stress is induced by a device, it demonstrates high test–retest and interobserver reliability for measurement of the lateral JSW [30–33]. Yet, these studies did not compare Rosenberg with the valgus stress radiograph while the Rosenberg is the most reliable weight-bearing radiograph to assess lateral cartilage wear, as this wear appears to be most prominent at the posterior aspect of the femoral condyle [13, 34].

One recent prospective study by Mortensen et al. performed a direct comparison between the Rosenberg and valgus stress radiograph in assessment of the lateral JSW in 73 patients prior to PKR or TKR [18]. Our results are in line with their findings; they demonstrated a 0.45–0.62 mm smaller lateral JSW on valgus stress compared to Rosenberg radiographs with a similar precision and strong correlation between the JSW measured on the Rosenberg and the valgus stress radiograph. Mortensen et al. concluded that the difference in lateral JSW is unlikely to be of impact in the patient selection for PKR and that the Rosenberg and valgus stress radiographs are interchangeable and. They advise the use of the Rosenberg radiograph only to save costs.

In addition to Mortensen et al., this study attempted to quantify the clinically relevant value of the valgus stress in addition to the Rosenberg radiograph when considering PKR in patients with medial osteoarthritis. First, the extent of the found difference in JSW between the Rosenberg and valgus stress radiograph was assessed. Although the calculated effect size was moderate (Cohen’s *d* = 0.53), the difference of 0.53–0.66 mm seems rather small when assessing the lateral JSW in a clinical setting [35]. On average, the valgus stress radiograph does not seem to detect a clinically relevant narrower lateral JSW compared to Rosenberg that could affect the decision for PKR. However, a more than 2 mm narrower JSW on the valgus stress compared to Rosenberg radiograph could be clinically relevant in the patient selection for PKR. In that case, the valgus stress radiograph measured a relevant difference in 10–11% of the patients. As the valgus stress radiograph measured a more than 2 mm narrower JSW mainly above a mean lateral JSW of more than 6 mm (11 out of 16 patients) rather than near the JSW of 5 mm, measurements at midJSW seem less able to distinguish between full thickness cartilage and cartilage wear. Therefore, the minJSW should be used in patient selection for PKR.

Moreover, the additional value of valgus stress radiograph was quantified as the ability to detect the presence of lateral JSN (JSW < 5 mm) in patients where JSN was absent on the Rosenberg radiograph (JSW > 5 mm) with a difference of > 2 mm between radiographs. Since a JSW of ≥ 5 mm is assumed to reflect full thickness cartilage on both tibial and femoral side, a JSW < 5 mm indicates lateral cartilage wear and is considered a contraindication for PKR. This study found an additional value of the valgus stress radiograph in 4–6% of the patients. The results translate into one patient every 17–25 patients, in which the valgus stress radiograph is of potential clinical value in addition to the Rosenberg in the patient selection for medial PKR. An interesting future perspective could be to determine specific patient, disease, or radiographic factors that could predict the cases in which the valgus stress radiograph has an additional value.

Besides the assessment of the lateral compartment cartilage, the valgus stress radiograph is deemed useful in preoperatively determining the varus deformity correctability [17]. Tashiro et al. and Zhang et al. have concluded that the valgus stress radiograph serves as a useful tool for assessing the correctability of the varus deformity [36, 37]. The postoperative alignment influences the outcomes of medial PKR. Substantial undercorrection can lead to increased bearing wear with aseptic loosening and specifically in mobile-bearing PKR to bearing dislocations [38, 39]. Limited studies have evaluated the usefulness of the valgus stress radiograph to assess correctability of the varus deformity preoperatively. Consistent with our results (94%), Waldstein et al. reported that the majority of knees (93%) with less than 10° varus deformity are corrected to the acceptable alignment of < 3° varus on valgus stress radiographs. Consequently, they concluded that the valgus stress radiograph provided no additional value, as an uncorrected varus deformity exceeding 10° is considered to be a contraindication for PKR [32]. While some studies propose a higher acceptable postoperative residual varus deformity of 4–7°, the percentage of knees correctable to an acceptable residual varus deformity could approach 100% [40–42]. Unlike JSW, the correctability of the varus deformity is adequately assessable during preoperative physical examination. Our results show that, in all the cases where the varus deformity correctability was specified, it was preoperatively redressable to a clinically acceptable axis. Notably, in none of our patients the decision for TKR was based on a fixed varus deformity, either preoperatively or intra-operatively. As Kreitz et al. demonstrated that adequate assessment of the varus deformity correctability can only be achieved after removing osteophytes perioperatively, the complete preoperative assessment in patients varus deformity remains a subject of debate [43].

The valgus stress radiograph has several disadvantages. First, the valgus stress radiograph is obtained using fluoro-guided positioning resulting in higher radiation doses than the Rosenberg radiograph. Second, there are extra costs for centers to acquire the equipment, as the reliability and reproducibility of the valgus stress radiograph increases with the use of standardized stress devices. In addition, there is a need to train personnel in performing the valgus stress radiograph adequately and is more time-consuming. These factors decrease its availability [33]. While probably of less importance in large, specialized centers, the availability of a valgus stress radiograph could be a problem in smaller centers.

Although representing clinical practice, the most important limitation of this study is its retrospective design. This results in radiographs obtained in an uncontrolled environment and by different radiographers, which impairs the quality and radio-anatomical alignment of the radiographs, and therefore the reliability of measuring the JSW. Since the valgus stress radiographs are fluoro-guided, the technique is more standardized and less prone to suboptimal alignment [13]. The Rosenberg radiographs, on other hand, are not fluoro-guided and alignment depends on many factors that could not be controlled in this study. Our data reflects a great variability in radio-anatomical alignment among the Rosenberg radiographs, of which only 39% were considered ‘optimal aligned’ (IMD < 1.7 mm) [22]. However, this variability in alignment created the opportunity to assess the effect of radio-anatomical alignment on the additional value of the valgus stress radiograph. As the alignment improves (IMD < 2.4 mm), the difference in JSW between the Rosenberg and valgus stress radiograph decreases to 0.39–0.55 mm and a > 2 mm lower JSW was only found in 4% of the patients. Moreover, additional value of the valgus stress radiograph to detect JSN with > 2 mm difference decreased to 3%. Thus, an improvement of radio-anatomical alignment on the Rosenberg results in a decrease of additional value of the valgus stress radiograph.

Second, a difference in JSW of > 2 mm between the Rosenberg and valgus stress might be considered as rather large to deem as clinically relevant. Dougados et al. stated that a change in JSW of > 0.5 mm in the hip can be regarded clinically relevant progression of OA. As this change was greater than the 95% CI of the measurement differences between raters, and therefore not related to measurement errors [44]. Applying similar methods, the upper limit in measurement difference between raters for this study was 1.9 mm. The best cutoff for a clinically relevant difference of JSW in decision-making for PKR needs further study.

Lastly, we based our results solely on the radiographic measurements rather than the impact on the surgeon’s preoperative decision-making. Although in 7 patients the surgeon stated that the decision for TKR was influenced by increased narrowing of the JSW on the valgus stress radiograph, in 6 of these patients the narrowing of the JSW was already measured on the Rosenberg (JSW < 5 mm). Therefore, in these patients, the decision for TKR could have been made based solely on the Rosenberg. A different study design (e.g., a case questionnaire among practicing knee surgeons) is required to investigate the impact of valgus stress radiographs on the surgeon’s decision-making.

## Conclusion

In conclusion, the results show that when assessing the lateral cartilage in the general population of patients with (suspected) medial knee osteoarthritis the valgus stress radiograph has a radiographically relevant value in addition to the Rosenberg in only 1 in 17–25 patients. Moreover, if the radio-anatomic alignment of the Rosenberg is optimal, the additional value of the valgus stress radiograph is negligible. For patients with (suspected) medial osteoarthritis, we recommend routine radiographic assessment with a Rosenberg radiograph and use an additional stress radiograph in case of discrepancy between clinical and radiological findings.

## Figures and Tables

**Fig. 4:**
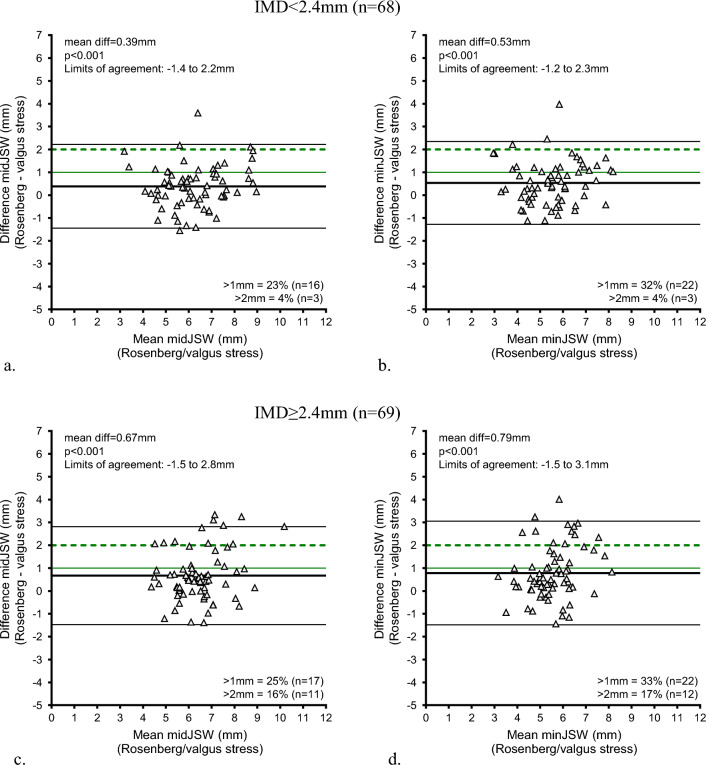
Level of agreement between Rosenberg and valgus stress radiographs divided by radio-anatomic alignment of the Rosenberg radiograph. Bland–Altman plots of agreement in JSW between Rosenberg and valgus stress radiographs at midJSW and minJSW for IMD < 2.4 mm (a, b) and IMD ≥ 2.4 (c, d) with mean difference, limits of agreement, and > 1 mm (green solid line) and > 2 mm (green dotted line) lower JSW measured on the valgus stress radiograph. *IMD* intermargin distance, *midJSW* midpoint joint space width, *minJSW* minimum joint space width, *JSW* joint space width

**Fig. 5:**
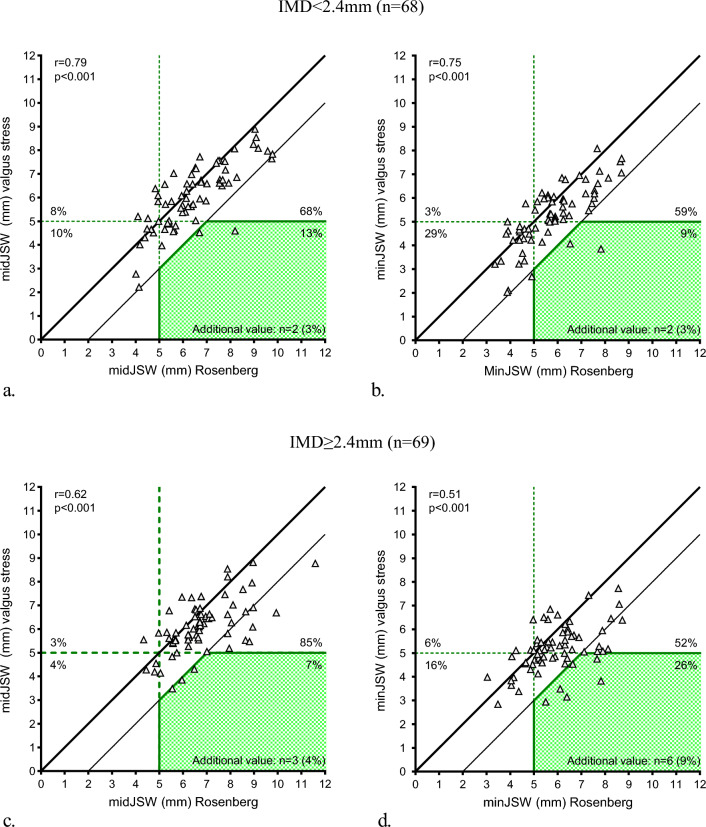
Relationships between Rosenberg and valgus stress radiographs divided by radio-anatomic alignment of the Rosenberg radiograph. Pearson correlation plots between Rosenberg and valgus stress radiographs at midJSW and minJSW for IMD < 2.4 mm (a, b) and IMD ≥ 2.4 (c, d) with the addition value (green shaded area) defined as: JSW ≥ 5 mm on the Rosenberg and < 5 mm on the valgus stress radiograph with > 2 mm difference (thin black line) between the measured values. *IMD* intermargin distance, *midJSW* midpoint joint space width, *minJSW* minimum joint space width, *JSW* joint space width

## Data Availability

The data are not shared openly due to information that could compromise the privacy of study participants. The data are available on request from the corresponding author, [SJL].

## References

[1] Wilson HA, Middleton R, Abram SGF, Smith S, Alvand A, Jackson WF (2019). Patient relevant outcomes of unicompartmental versus total knee replacement: systematic review and meta-analysis. BMJ.

[2] Beard DJ, Davies LJ, Cook JA, MacLennan G, Price A, Kent S (2019). The clinical and cost-effectiveness of total versus partial knee replacement in patients with medial compartment osteoarthritis (TOPKAT): 5-year outcomes of a randomised controlled trial. Lancet (London, England).

[3] Migliorini F, Tingart M, Niewiera M, Rath B, Eschweiler J (2019). Unicompartmental versus total knee arthroplasty for knee osteoarthritis. Eur J Orthop Surg Traumatol.

[4] Murray DW, Liddle AD, Dodd CA, Pandit H (2015). Unicompartmental knee arthroplasty: is the glass half full or half empty?. Bone Jt J.

[5] Hamilton TW, Rizkalla JM, Kontochristos L, Marks BE, Mellon SJ, Dodd CAF (2017). The interaction of caseload and usage in determining outcomes of unicompartmental knee arthroplasty: a meta-analysis. J Arthroplast.

[6] Liddle AD, Pandit H, Judge A, Murray DW (2016). Effect of surgical caseload on revision rate following total and unicompartmental knee replacement. J Bone Jt Surg Am.

[7] Goodfellow JW, O'Connor JJ, Murray DW (2010). A critique of revision rate as an outcome measure: re-interpretation of knee joint registry data. J Bone Jt Surg Br.

[8] van Oost I, Koenraadt KLM, van Steenbergen LN, Bolder SBT, van Geenen RCI (2020). Higher risk of revision for partial knee replacements in low absolute volume hospitals: data from 18,134 partial knee replacements in the Dutch arthroplasty register. Acta Orthop.

[9] Kozinn SC, Scott R (1989). Unicondylar knee arthroplasty. J Bone Jt Surg Am.

[10] Pandit H, Jenkins C, Gill HS, Smith G, Price AJ, Dodd CA (2011). Unnecessary contraindications for mobile-bearing unicompartmental knee replacement. J Bone Jt Surg Br.

[11] Goodfellow J, O'Connor J, Murray DW (2002). The Oxford meniscal unicompartmental knee. J Knee Surg.

[12] Buckland-Wright JC, Macfarlane DG, Lynch JA, Jasani MK, Bradshaw CR (1995). Joint space width measures cartilage thickness in osteoarthritis of the knee: high resolution plain film and double contrast macroradiographic investigation. Ann Rheum Dis.

[13] Buckland-Wright C (2006). Which radiographic techniques should we use for research and clinical practice?. Best Pract Res Clin Rheumatol.

[14] Rosenberg TD, Paulos LE, Parker RD, Coward DB, Scott SM (1988). The forty-five-degree posteroanterior flexion weight-bearing radiograph of the knee. J Bone Jt Surg Am.

[15] Mukherjee K, Pandit H, Dodd CA, Ostlere S, Murray DW (2008). The Oxford unicompartmental knee arthroplasty: a radiological perspective. Clin Radiol.

[16] Hamilton TW, Pandit HG, Lombardi AV, Adams JB, Oosthuizen CR, Clave A (2016). Radiological decision aid to determine suitability for medial unicompartmental knee arthroplasty: development and preliminary validation. Bone Jt J.

[17] Oosthuizen CR, Burger S, Vermaak DP, Goldschmidt P, Spangenberg R (2015). The X-ray knee instability and degenerative score (X-KIDS) to determine the preference for a partial or a total knee arthroplasty (PKA/TKA). South Afr Orthop J (SAOJ).

[18] Mortensen JF, Kappel A, Rasmussen LE, Ostgaard SE, Odgaard A (2021). The Rosenberg view and coronal stress radiographs give similar measurements of articular cartilage height in knees with osteoarthritis. Arch Orthop Trauma Surg.

[19] Dupuis DE, Beynnon BD, Richard MJ, Novotny JE, Skelly JM, Cooper SM (2003). Precision and accuracy of joint space width measurements of the medial compartment of the knee using standardized MTP semi-flexed radiographs. Osteoarthr Cartil.

[20] Mehta N, Duryea J, Badger GJ, Akelman MR, Jones MH, Spindler KP (2017). Comparison of 2 radiographic techniques for measurement of tibiofemoral joint space width. Orthop J Sports Med.

[21] Mazzuca SA, Le Graverand MPH, Vignon E, Hunter DJ, Jackson CG, Kraus VB (2008). Performance of a non-fluoroscopically assisted substitute for the Lyon schuss knee radiograph: quality and reproducibility of positioning and sensitivity to joint space narrowing in osteoarthritic knees. Osteoarthr Cartil.

[22] Vignon E, Brandt KD, Mercier C, Hochberg M, Hunter D, Mazzuca S (2010). Alignment of the medial tibial plateau affects the rate of joint space narrowing in the osteoarthritic knee. Osteoarthr Cartil.

[23] Bland JM, Altman DG (1986). Statistical methods for assessing agreement between two methods of clinical measurement. Lancet (London, England).

[24] Chan YH (2003). Biostatistics 104: correlational analysis. Singap Med J.

[25] Gibson PH, Goodfellow JW (1986). Stress radiography in degenerative arthritis of the knee. J Bone Jt Surg Br.

[26] Arifin WN (2018). A web-based sample size calculator for reliability studies. Educ Med J.

[27] Koo TK, Li MY (2016). A guideline of selecting and reporting intraclass correlation coefficients for reliability research. J Chiropr Med.

[28] Price AJ, Svard U (2011). A second decade lifetable survival analysis of the Oxford unicompartmental knee arthroplasty. Clin Orthop Relat Res.

[29] Vignon E, Conrozier T, Piperno M, Richard S, Carrillon Y, Fantino O (1999). Radiographic assessment of hip and knee osteoarthritis. Recommendations: recommended guidelines. Osteoarthr Cartil.

[30] Eriksson K, Sadr-Azodi O, Singh C, Osti L, Bartlett J (2010). Stress radiography for osteoarthritis of the knee: a new technique. Knee Surg Sports Traumatol, Arthrosc Off J ESSKA.

[31] Tallroth K, Lindholm TS (1987). Stress radiographs in the evaluation of degenerative femorotibial joint disease. Skelet Radiol.

[32] Waldstein W, Bou Monsef J, Buckup J, Boettner F (2013). The value of valgus stress radiographs in the workup for medial unicompartmental arthritis. Clin Orthop Relat Res.

[33] Koppens D, Sorensen OG, Munk S, Rytter S, Larsen SKA, Stilling M (2019). The lateral joint space width can be measured reliably with Telos valgus stress radiography in medial knee osteoarthritis. Skelet Radiol.

[34] Mazzuca SA, Brandt KD, Buckwalter KA, Lane KA, Katz BP (2002). Field test of the reproducibility of the semiflexed metatarsophalangeal view in repeated radiographic examinations of subjects with osteoarthritis of the knee. Arthritis Rheum.

[35] Cohen J (2013). Statistical power analysis for the behavioral sciences.

[36] Tashiro Y, Matsuda S, Okazaki K, Mizu-Uchi H, Kuwashima U, Iwamoto Y (2014). The coronal alignment after medial unicompartmental knee arthroplasty can be predicted: usefulness of full-length valgus stress radiography for evaluating correctability. Knee Surg Sports Traumatol Arthrosc Off J ESSKA.

[37] Zhang Q, Yue J, Wang W, Chen Y, Zhao Q, Guo W (2018). FTFA change under valgus stress force radiography is useful for evaluating the correctability of intra-articular varus deformity in UKA. Arch Orthop Trauma Surg.

[38] Hernigou P, Deschamps G (2004). Alignment influences wear in the knee after medial unicompartmental arthroplasty. Clin Orthop Relat Res.

[39] van der List JP, Zuiderbaan HA, Pearle AD (2016). Why do medial unicompartmental knee arthroplasties fail today?. J Arthroplasty.

[40] Zuiderbaan HA, van der List JP, Chawla H, Khamaisy S, Thein R, Pearle AD (2016). Predictors of subjective outcome after medial unicompartmental knee arthroplasty. J Arthroplast.

[41] Vasso M, Del Regno C, D'Amelio A, Viggiano D, Corona K, Schiavone PA (2015). Minor varus alignment provides better results than neutral alignment in medial UKA. Knee.

[42] van der List JP, Chawla H, Villa JC, Pearle AD (2016). Different optimal alignment but equivalent functional outcomes in medial and lateral unicompartmental knee arthroplasty. Knee.

[43] Kreitz TM, Maltenfort MG, Lonner JH (2015). The valgus stress radiograph does not determine the full extent of correction of deformity prior to medial unicompartmental knee arthroplasty. J Arthroplast.

[44] Dougados M (2004). Monitoring osteoarthritis progression and therapy11supported by Procter & Gamble pharmaceuticals, Mason, OH. Osteoarthr Cartil.

